# Governing AI in Southeast Asia: ASEAN’s way forward

**DOI:** 10.3389/frai.2024.1411838

**Published:** 2024-08-30

**Authors:** Bama Andika Putra

**Affiliations:** ^1^School of Sociology, Politics, and International Studies, University of Bristol, Bristol, United Kingdom; ^2^Department of International Relations, Universitas Hasanuddin, Makassar, Indonesia

**Keywords:** ASEAN, Southeast Asia, artificial intelligence, AI governance, governance

## Abstract

Despite the rapid development of AI, ASEAN has not been able to devise a regional governance framework to address relevant existing and future challenges. This is concerning, considering the potential of AI to accelerate GDP among ASEAN member states in the coming years. This qualitative inquiry discusses AI governance in Southeast Asia in the past 5 years and what regulatory policies ASEAN can explore to better modulate its use among its member states. It considers the unique political landscape of the region, defined by the adoption of unique norms such as non-interference and priority over dialog, commonly termed the ASEAN Way. The following measures are concluded as potential regional governance frameworks: (1) Elevation of the topic’s importance in ASEAN’s intra and inter-regional forums to formulate collective regional agreements on AI, (2) adoption of AI governance measures in the field of education, specifically, reskilling and upskilling strategies to respond to future transformation of the working landscape, and (3) establishment of an ASEAN working group to bridge knowledge gaps among member states, caused by the disparity of AI-readiness in the region.

## 1 Introduction

For member states of the Association of Southeast Asian Nations (ASEAN), the importance of artificial intelligence (AI) has gained traction among Southeast Asian policymakers. ASEAN defines AI as “…an engineered or machine-based system that can, for a given set of objectives, generate outputs as predictions, recommendations, or decisions influencing real or virtual environments” (ASEAN, 2024b, p. 9). The region holds vast market potential, as the digital economy alone has the prospective of achieving revenues of up to USD 218 billion in transactions in 2023 (Wang, 2024), further highlighting the trend of the digital economy and the integration of digitalization efforts in vast creative economic fields in the region. By 2030, as shown in Table 1, Kearney Analysis predicted that AI would contribute 10 to 18% to the region’s GDP (Kearney, 2020). The disparate impact of AI seen in Table 1 is due to the different economic conditions that Southeast Asian states are currently facing. Countries like Singapore, Malaysia, and Indonesia, with more advanced economies, are predicted to utilize AI to integrate modernization within their borders further. Meanwhile, CMLV (Cambodia, Myanmar, Laos, Vietnam), known to be the ASEAN member states with a considerable development gap with other members, are expected to integrate AI technology at a more macro-level. Southeast Asia also holds considerable human resources as both a source of market and a driving force to AI usage in day-to-day activities, having hosted approximately 700 million people, leading ASEAN to adopt a 2025 vision of becoming a “leading digital community and economic bloc” (Putra, 2022; ASEAN, 2024a).

**Table 1:** AI impact on Southeast Asian GDP (% of 2030 GDP).

ASEAN member state	Expected AI-value on GDP	Percentage of 2030 GDP
Singapore	USD 110 Billion	18%
Malaysia	USD 115 Billion	14%
Thailand	USD 117 Billion	13%
Indonesia	USD 366 Billion	12%
Philippines	USD 92 Billion	12%
Vietnam	USD 109 Billion	12%
Brunei, Cambodia, Laos, Myanmar	USD 41 Billion	10%
Southeast Asian States	USD 950 Billion	13%

Source: Analysis of Kearney (2020).

However, unlike other regions, such as Europe, Southeast Asia has struggled to effectively govern the use of AI. ASEAN hosts member states with diverse political systems and contrasting rights of online information access. This is coupled with issues related to censorship, disinformation, and the intensification of social media usage among the younger demographic population. So, when member states such as Singapore have displayed innovation and readiness through domestic regulatory frameworks governing AI (Fitriani, 2024), others are still in their early development plans (Isono and Prilliadi, 2023). Regionally, ASEAN member states have been limited in a unified perception of AI, having only published a guide on AI governance and ethics in 2024, without any imposed regulations on its usage.

ASEAN’s unique political context exacerbates the lack of a regional approach to AI. Founded in 1967, ASEAN has been the primary regional organization that constructs and disseminates norms for its members in the Southeast Asian region. Unlike organizations such as the European Union (EU), ASEAN is not a supranational organization that can impose regulations on its members. This is due to its core values, which include non-interference and non-intervention from foreign dictates, as well as a consensus decision-making system. Consequently, this system continues to be a challenge, considering the differences in national interests posed by the diverse political systems evident in Southeast Asia, ranging from an absolute monarchy to a multiparty democracy to a socialist republic.

The disparities in AI readiness and governance in Southeast Asia are concerning. In other parts of the world, AI has been utilized across banking and accounting sectors to allow greater customer service experience and detect conducts of fraud (Jeong et al., 2023; Saleem et al., 2023; Tanbour and Nour, 2024). In education, AIs have been used to disseminate a better teaching and learning process for students across different educational stages (Wang et al., 2023). AI’s usage has also assisted in diagnosing and treating diseases in the healthcare system (Shi and Zhao, 2018). Due to its vast potential to transcend different sectors, AI’s governance in developed countries has been facilitated with nuanced policies encouraging greater AI development (Gonzales, 2023). For example, the EU’s AI legislation has acted as the frontier of a regional legal framework on AI in the past decade (Almada and Radu, 2024). Consequently, this raises concerns for ASEAN’s AI governance in the diverse Southeast Asian region.

This qualitative inquiry discusses AI governance in Southeast Asia and the regulatory policies that ASEAN can explore to better regulate its use among its member states. It considers the unique political landscape of the region, defined by the adoption of unique norms such as non-interference and priority over dialog, commonly termed the ASEAN Way. This study utilizes secondary data on ASEAN member states’ AI governing in the past 5 years, gathered from accessible online documents. It offers the author’s perspective on the way forward, incorporating the importance of accelerating AI’s use across different fields but balanced with considering the region’s unique social nuances and sensitivity to legally enforcing policies. The following sections will start by arguing what advances have been made in the region, followed by the authors’ recommendations for ASEAN in the future.

## 2 AI disparities in Southeast Asia: status quo policies and persistent issues

How have Southeast Asian states utilized AI in a region of technological disparities? Singapore is undoubtedly showcasing itself as Southeast Asia’s AI hub (Fitriani, 2024). It was the first ASEAN member state to introduce a National AI strategy in 2019 and has, since then, rapidly invested in the use of AI among its population. Vietnam recently introduced PhoGPT, inspired by the Vietnamese noodle Pho, as a ChatGPT alternative using the Vietnamese language (Wang, 2024). Thailand has intensified its AI usage in the state’s transportation affairs, while Indonesia has recently focused on the agricultural and health sectors (Fitriani, 2024).

Diversities to AI readiness can also be seen in the different responses to the EU’s General Data Protection Regulation (GDPR). As past studies suggest, machine learning systems are fueled by data, and personal data helps build construct algorithmic models (Sartor, 2020; Baig, 2023). ASEAN member states are currently reviewing their data protection laws. Singapore, the Philippines, and Thailand have introduced laws that partially align (not in whole) toward the requirements since 2020. Meanwhile, in 2022, Indonesia issued the Personal Data Protection Bill, the first law in the country that governs personal data protection in a comprehensive sense (THALES, 2024). Nevertheless, it is essential to note that all Southeast Asian states had some form of data and privacy protection law in place prior to introducing the GDPR in 2018 (Gan, 2018), albeit not to the standards set under the GDPR. Personal data and the lack of understanding toward the consequences of AI have resulted to a slow progress in the introduction of new regulatory frameworks.

The problem of AI disparities is well documented in the Oxford Insights study on AI Readiness 2022 in Table 2. The region hosts one of the highest AI-ready countries in the world, with Singapore ranked second. Still, at the same time, it also consists of significantly low-ranked states, with Myanmar, Laos, and Cambodia ranked above 126.

**Table 2:** Southeast Asian AI-readiness index (Government) 2022.

Country	Total score	Global position
Singapore	84.1	2
Malaysia	67.4	29
Thailand	64.6	31
Indonesia	60.9	43
Philippines	55.4	54
Viet Nam	54.0	55
Brunei Darussalam	48.1	67
Myanmar	32.5	126
Laos	31.7	129
Cambodia	31.2	132

Source: Oxford Insights (2023).

Consequently, it has not been easy for ASEAN to assemble a suitable regional governance approach vis-à-vis this imbalance. The differences in the ASEAN member states’ AI readiness lead to different policy focuses across Southeast Asian states, thus establishing flexible approaches in regional AI governance. Chiang also reported that, at the bare minimum, ASEAN member states even have dissimilar concerns over non-traditional security threats related to AI (such as cybersecurity, fake news, and disinformation), solidifying the challenge of regional approaches (Chiang, 2024). For example, the non-democratic ASEAN member states tend to echo the importance of fake news and disinformation that have undermined state authority; meanwhile, democratic ASEAN member states have been vocal about the threat of cybersecurity undermining government operations. The most plausible regional action has been the publication of the ASEAN Guide to AI Governance and Ethics 2024, setting out a non-binding recommendation for the use of AI for government and non-government stakeholders in the region. The aim is to establish the responsible use of modern AI technologies across Southeast Asian states.

This perspective identifies two issues with ASEAN’s AI guide. First, perhaps clear with the naming of the document as a “guide,” this document will not supersede laws. It will remain a guideline for government and non-government stakeholders on the ethically responsible usage of AI. Still, it will not be able to impose sanctions if a member state decides to take different routes of action in developing its AI technologies. Second, ASEAN has been vocal about not siding with any power (including the EU) in determining the trajectory of its AI development (Wang, 2024). It has not leaned toward the EU’s AI guidance, as ASEAN does not wish to impose a strict regulatory framework for its member states. This posture further solidifies the ASEAN way of its policies, which are not dictated by great powers, and how Southeast Asian states prioritize self-determination.

Nevertheless, ASEAN’s shallow regional governance on AI has caused some lingering issues to persist. Public opinion on AI is a critical concern. There is unease about how AI is used in developing Southeast Asian economies, with fears of job displacements, ethical considerations, and a lack of disclosed information on when AI is being used. As the nature of AI usage in the region is relatively new, users have not been adequately informed on the circumstances under which AI is operated, thus leading to a negative public trust toward AI. Ethical concerns are also relevant to the Southeast Asian context. In the past, there have been instances in which AI algorithms discriminated against certain demographic features in the process of job applications (ASEAN, 2024a). If not properly constructed, AI systems could sustain discriminatory impacts on specific groups in societies. This is concerning for Southeast Asian states, considering their history of colonization, discrimination, and decades of human rights violations.

An equally important concern is the inadequate cybersecurity measures to counter fake news and disinformation. ASEAN has outlined several regulations that help Southeast Asian states counter this issue (ASEAN, 2023; Chongkittavorn, 2023; Martinus, 2023). However, there is a lack of connection between fake news and AI technological advancements in the region. Thus, there are fears that AI systems could be vulnerable to cyberattacks, especially considering Southeast Asian AI is at its initial development stage for most countries. This, however, does not apply to Singapore with its introduced “Protection from Online Falsehoods and Manipulation Act” of 2019. This grants Singaporean authorities fast-checking capabilities, censorship of both websites and social media platforms, and even the possibility of criminal charges (Vaswani, 2019).

These regional challenges, coupled with the unique political landscape of Southeast Asian states and the ASEAN Way of collaborative regional approaches, are convoluted for AI development. It remains a discourse that has been partially assessed in the past literatures without exploring potential solutions to how regional approaches are able to curtail the challenges faced. The following section will recommend ASEAN’s AI regional governance by elevating its importance, education-oriented governance measures, and establishing an ASEAN working group. In doing so, it will combine academic discourses of AI governance with the author’s original perspectives.

## 3 ASEAN’s way forward: importance elevation, education-oriented governance, and an ASEAN AI working group

There has not been a shortage of studies accessing ASEAN’s AI dynamics. Before adopting the ASEAN AI guidelines, studies have focused on the importance of a regional policy framework to be adopted by the regional organization (Chitturu et al., 2017). However, most studies have agreed that the fast-paced development of AI needs to be met by an ASEAN-centered strategy to mitigate risks while simultaneously capitalizing on its benefits (Marsan, 2021). However, two things must be highlighted when formulating ASEAN’s way forward regarding regional AI governance in Southeast Asia. First, ASEAN has no right to pass laws that supersede national policies. Second, Southeast Asia’s AI landscape faces similar opportunities but contrasting challenges due to the unique political features in the region.

This perspective piece argues that ASEAN needs a comprehensive policy framework vis-à-vis the fast-paced development of AI in the region. In doing so, policies will identify areas that may be susceptible to disagreements (such as with the definitional differences on what constitutes non-traditional security threats), consider the importance of ethics for the Southeast Asia demography, and engage multiple stakeholders (government and non-government), as well as measures to counter the possible risks that may arise. The author also echoes the thoughts introduced by Fitriani in 2024, in which the development of AI-related policies must consist of intensive monitoring and adaptations (Fitriani, 2024). Nevertheless, striking the correct balance between under and over-regulation may be difficult in the context of ASEAN. The regional organization needs to be wary of the diverse norms relevant under the ASEAN context and the sensitivity of member states regarding specific topics thought to undermine a member state’s sovereignty.

Therefore, the first recommendation is to elevate AI’s importance in ASEAN forums. In the past, ASEAN member states were also conflicted in responding to the issues of haze pollution and handling the South China Sea. In the case of haze pollution, Singapore, Malaysia, and Indonesia have been in contestation with one another regarding what measures need to be adopted and who should be most responsible for the issue (Greenpeace, 2019; ASEAN, 2024b). In the South China Sea, there have been divided opinions with ASEAN on what measures should collectively be taken vis-à-vis China’s aggression at sea (Odgaard, 2003; Storey, 2018). Nevertheless, in both cases, ASEAN member states have found common ground with adopting the ASEAN Agreement on Transboundary Haze Pollution and the Declaration on the Conduct of Parties in the South China Sea. Though both cannot replace national-level regulations, the collective agreement has been able to properly guide ASEAN states to take a typical path in responding to issues that have caused disagreements.

Elevating the topic of AI in Southeast Asia can be the foundation for a collective agreement to regulate the proper use of AI in the region. Despite the two cases previously mentioned being in the form of an agreement (thus, not legally binding), the agreements have been perceived as a milestone in ASEAN’s governance of environmental issues and conflict management in Southeast Asia (Buszynski, 2003; Greenpeace, 2023). However, this was only made possible due to the constant reference of the problems in ASEAN intra and inter-regional forums. Discussing AI’s wider regional opportunities and challenges in ASEAN’s extra-regional forums, such as the ASEAN Regional Forum, ASEAN+3, and the East Asian Summit, may create a more vital urgency to adopt collective regional measures in responding to AI’s fast-paced development.

Second, this study believes that education-oriented governance through reskilling and upskilling may solve the lack of public awareness and digital literacy related to AI. Part of the problem has been the lack of public awareness about what AI usage entails in one’s daily life. Consequently, public perception has been mixed, with some fearing possible loss of jobs due to the adoption of AI-based technologies. Working with government and non-government stakeholders, ASEAN needs to develop educational approaches better to understand AI technology and its consequences for the workforce. This could include upskilling and reskilling strategies collectively adopted among Southeast Asian states to counter possible disturbances to Southeast Asia’s employment landscape. Doing so would advance the chances of developing skills that are irreplicable amid the presence of automated systems within a corporation, allowing for a synergy between manual labor and AI technologies to coexist. First, ASEAN Education Ministers’ Meeting to develop an intra-regional approach to AI-related educational reskilling and upskilling efforts. Second, the ASEAN Plus Three Education Ministers Meeting would include education ministers from South Korea, Japan, and China. This exposes ASEAN to East Asia’s best practices in AI-relevant education.

Last is establishing a working group within ASEAN tasked to guide member states in developing AI governance. ASEAN’s 2024 AI guide was considered a milestone in which ASEAN recognized the importance of AI for the region’s development. However, as some ASEAN member states do not have the capacity and technical expertise to develop their AI governance further, there is a high probability that a more significant disparity gap will be created in the coming years. A working group is needed to ensure the region can equally enjoy access to the operational implementation of AI governance. This working group can provide ASEAN member states the proper assistance to implement the 2024 ASEAN AI guide provisions and continuously consult with relevant stakeholders for greater regional AI governance. Considering the different AI-readiness levels of ASEAN member states, a working group allows for a focused recommendation toward each individual member by catering to the unique political landscapes that may require different approaches in regulatory frameworks. As in the operationalization of the ASEAN Indo-Pacific Outlook, working groups provide technical assistance that allows ASEAN to move away from a simple normative agreement in countering a rising issue.

## 4 Conclusion

The potential of AI in Southeast Asia is met with contemporary challenges that have started to surface in the region. ASEAN has not been able to adopt a decisive strategy in AI governance, partly due to the disparity of AI readiness in Southeast Asia. Singapore is considered the only ASEAN member state ready to respond to the challenges and opportunities posed by AI. Meanwhile, the other nine member states are either in the middle or ranked low in their AI readiness index. This is concerning, considering that AI holds great potential in advancing Southeast Asia’s GDP in the coming years.

Three recommendations are put forward. The first is to elevate the AI topic in ASEAN intra- and inter-regional forums. ASEAN still has the capacity to adopt policies that could guide state policymaking in the region. It does so by adopting agreements only if an issue is perceived as an essential challenge that necessitates regional resolutions. Elevating the AI topic in ASEAN allows regional agreements to be concluded despite the lingering disparity of AI readiness in Southeast Asia. Equally essential for the region is an education-oriented governance approach focusing on reskilling and upskilling. As elaborated in this study, public perception and awareness of AI is necessary. Fears of a transforming working landscape have severely affected AI acceptance among the Southeast Asian population. This challenge can be countered by reskilling and upskilling skills relevant to AI in the region, allowing for greater working adaptability. Last, a working group for AI governance in ASEAN is recommended in order to address the operational and technical needs of ASEAN member states and formulate national governance approaches.

## Data Availability

The original contributions presented in the study are included in the article/supplementary material, further inquiries can be directed to the corresponding author.
